# Actinobacillosis1Read before the Pennsylvania State Veterinary Medical Association’s Annual Meeting, March 3 and 4, 1903.

**Published:** 1903-05

**Authors:** S. J. J. Harger

**Affiliations:** Philadelphia, Pa.


### SELECTIONS.

### ACTINOBACILLOSIS.[1]

By S. J. J. Harger, V.M.D.,
PHILADELPHIA, PA.

In the summer of 1900 an epizootic occurred among the cattle of the Argentine Republic, which, in its clinical aspect, resembled actinomycosis. The latter disease was known to exist there since 1887, affecting from $\frac{1}{2}$ to 1 per cent. of the animals in some localities. The disease in question was investigated by Lignieres and Spitz, of the National Bacteriologic Institute of Buenos Ayres, and by them named actinobacillosis.

During this epizootic, in some localities from 40 to 50 per cent. of the animals were affected; the improved breeds more so than the native stock, and in rare cases sheep also; in one instance a calf one month old was affected.

*Clinical Study.* Nearly all the tissues of the body were the seat of lesions; the skin, subcutaneous tissue, lymphatic and salivary glands, pharynx, lungs, and bones. The skin of the throat and neck was most frequently affected. The subcutaneous tissue became the seat of a hard, fibrous tumor, as large as a fist and larger, which fluctuated, and, finally, the abscess broke. The orifice became surrounded with exuberant granulations, and the tumor remained in this state indefinitely; sometimes it became absorbed. These abscesses, which were usually multiple, were found at the base of the ear, the throat, upper jaw, intermaxillary space, along the mastoid humeralis muscle, ribs, flanks, sheath, and inside of the thighs. Sometimes the disease manifests itself as a subcutaneous hyperplasia between the hock and the hoof, resembling elephantiasis, and accompanied by multiple abscess and grayish fungoid ulcerations. The lymphatic glands, contrary to the lesions of actinomycosis, around the head and neck may become enlarged and suppurate primarily or from infection by the neighboring lesions. Invasion of the tongue resembles "wooden tongue" of actinomycosis; it becomes hypertrophied, studded with nodules and ulcerations, deglutition becomes impossible, and the animal dies from starvation. In the pharynx the lesions consist of polypoid tumors; in the mammary

[1] Read before the Pennsylvania State Veterinary Medical Association's Annual Meeting, March 3 and 4, 1903.

glands of chronic interstitial mammitis, with abscesses. In the lungs are found nodules of various sizes enclosing the characteristic pus; in the upper and lower jaws, hypertrophy and arealation of the bone tissue with multiple abscesses. The disease shows all the peculiarities of location of actinomycosis, and in 80 per cent. the neck is the seat of the lesion. This epizootic in the Argentine was more destructive than foot-and-mouth disease which had existed some months before. The disease was usually introduced into a herd by newly purchased animals. Of those affected, some were killed, and others were sent to the abattoirs. From 15 to 50 per cent. of a herd became affected. Such a rapid spread of the disease is not the rule, since numerous sporadic cases are found, but at a given time some undetermined influence may increase the virulence of the pathogenic agent. Contact with the diseased animal, or forage, or other objects soiled with the pus, is necessary to transmit the contagion.

*Bacteriology.* The pus from the abscess is milky-white or slightly grayish in tint, thick, viscid, odorless, and contains numerous small grumous masses, the latter varying in size from a pin-point to a pin-head, reveals under the microscope one or more tufts with numerous club-shaped elements somewhat simulating the ray fungus of actinomycosis. It is differentiated from the latter by *not taking the stain of Gram.* Save in the abscess on the jaws, the pus does not contain the yellow granules seen in lump-jaw pus.

Pus drawn from a closed aperture rarely gives any culture upon ordinary media unless it is first ground in a sterilized mortar. In twenty-four hours, at 37° C., numerous small, bluish, translucid colonies will grow upon gelose, which is the best culture medium. Bouillon, milk, and blood serum of the horse may also be used. Potato will not grow colonies unless it is first rendered alkaline.

The specific agent of the cultures, the actinobacillis, not much larger than the bacillus of chicken cholera, measures $\frac{1}{25} \times 4\ \mu$. It contains and forms no spores. The first cultures are purely bacillary; during successive inoculations they assume the form of a cocco-bacillus, a diplococcus; or, in bouillon serum, a strepto-bacillus. It stains readily with aniline, phenicated, fuchsin, and acid violet, but not with the method of Gram.

This bacillus possesses very little resistance to physical and antiseptic agents and to desiccation, and loses its virulence in about two months. The cultures contain a toxin, actinobacillin, demonstrated by inoculating ruminants and other animals with a glycerin extract. It causes febrile reaction, 1 to $2\frac{1}{2}$ per cent., trembling, suspension of rumination, and loss of appetite toward the fifth hour. The results obtained from injections for diagnostic purposes are inconstant.

The actinobacillus being proved to be the specific pathogenic agent, Lignieres and Spitz determined the relation between the bacillus and the club-tufts found in the pus. This they did by injecting cultures of the bacillus under the skin, and examining with the microscope the local lesions in their successive stages to the maturing of the abscess. During the progress of the lesions the bacilli gradually disappeared; some massed themselves in the polynuclear leucocytes, and by a process of germination ultimately developed into the tufts. The experimenters could see the bacillary origin of the youngest tufts as well as the successive forms leading to the matured ones. This is a remarkable transformation of a microbe in the animal tissues, and granting that it be so, and reasoning by analogy, we may conclude that the actinomyces of actinomycosis is only an ultimate product and not the primary cause.

*Inoculations.* Intravenous injections of pure cultures in the horse, dog, cat, and pig produce toxæmia, and sometimes death. Subcutaneous inoculations cause local abscess, but no characteristic lesion of the disease; in the sheep the pus is characteristic, but the parts heal in a few weeks. In the ox toxic symptoms of intravenous injections are most marked. Subcutaneous injection of a pure gelose culture proves the specific rôle of the bacillus by producing a local phlegmon identical with that of the spontaneous disease. As in the latter, the abscess which attains the size of a fist, has no tendency to heal if left to itself. In one case the infection became metastatic; abscesses were found in the muscle of the thigh, the sacral, sublumbar, and mediastinal lymphatic glands, and the lungs. Cultures from this pus showed the presence of the bacillus. Ingestion of the pus and of pure cultures was always negative.

But little is known of the mode of infection. The bacillus has not been found on any of the forage. As in actinomycosis, the possible channel of entrance is the digestive tract, through abrasions in the mucous membrane. In the outbreak in the Argentine the infection was attributed to abrasions in the mouth left by foot-and-mouth disease.

The prophylaxis and treatment are those of a contagious disease. An infected animal in a herd may under certain conditions become a source of contagion. The healthy animals should be placed upon non-infected pastures, and removed from the diseased. The latter may be treated or slaughtered. The meat, where the diseased parts are removed, can be used for consumption, and this may be the more economic course.

Iodide of potassium, it is said, is an excellent specific, and under its prolonged treatment the lesions gradually disappear, except when the bones are affected 10 to 12 grammes are given daily, or 1 c.c. of a 10 per cent. solution, and 1 c.c. of Lugol's solution are injected at different points into the thickness of the tumor. Abscesses should be opened and curetted, but this operation alone is not sufficient, and is only a useful adjuvant to the iodide of potash.

This disease is recidivant, a first attack not conferring immunity. Bovines that have recovered from or carry lesions of the disease are even more sensitive to pure cultures than those never affected. A vaccine is, therefore, out of the question. A serum obtained from a mare inoculated with large doses of pure cultures has clearly shown itself to be preventive in guinea-pigs inoculated with fatal doses. This requires further research.

The disease has certain points of analogy with actinomycosis. The seat and the macroscopic characters of the lesion, its therapeutic reaction to iodide of potash and the club-tufts found in the pus. The points of difference are: The pus does not contain yellow granulations; the tufts do not take the stain of Gram. A specific bacillus has been found, and the disease appears to be more contagious. Without accepting conclusions prematurely, it appears that the work was done by men of the highest standing in the bacteriologic world. Nocard has identified the disease in Europe. If it exists in this country, it no doubt has been confounded with actinomycosis, and to make observations is interesting. Since no cattle are imported from South America into this country, there is no danger of contagion from that source.

